# Efficacy of 5×5 accelerated versus conventional repetitive transcranial magnetic stimulation (rTMS) for treatment-resistant depression

**DOI:** 10.21203/rs.3.rs-7377114/v1

**Published:** 2025-08-19

**Authors:** Michael Apostol, Thomas Valles, Juliana Corlier, Michael Leuchter, Alexander Young, Hewa Artin, Ralph Koek, Evan Einstein, Scott Wilke, Reza Tadayonnejad, Hanadi Oughli, Thomas Strouse, Aaron Slan, Margaret Distler, Dustin DeYoung, Nathaniel Ginder, David Krantz, Andrew Leuchter

**Affiliations:** University of California, Los Angeles; University of California, Los Angeles; UCLA; UCLA; UCLA

## Abstract

Accelerated repetitive transcranial magnetic stimulation (rTMS) is an effective treatment for Major Depressive Disorder (MDD), with an FDA-cleared protocol utilizing 10 sessions of prolonged intermittent Theta Burst Stimulation (piTBS) per day for five days. However, it remains unclear how many sessions and pulses are necessary to achieve benefit. We examined efficacy of five stimulation sessions per day for five days (“5×5”) of either piTBS or individualized, electroencephalogram-based “resonant frequency” (RF) stimulation. We compared MDD symptom improvement (PHQ-9) between two groups: 1) accelerated patients (N = 40) undergoing 5×5 treatment (patients received piTBS or RF rTMS) and 2) conventional patients (N = 135) receiving standard once daily rTMS. Both protocols ameliorated MDD symptoms and there were no statistically significant differences in depression symptom changes (p = .07) between the accelerated 5×5 protocol (mean PHQ-9: pre-rTMS = 17.68, post-rTMS = 10.98) and conventional once-daily treatment (mean PHQ-9: pre-rTMS = 17.83, post-rTMS = 8.97). There was no significant difference in improvement between the piTBS and RF 5×5 protocols (p = 0.9). A PHQ-9 median split of 5×5 patients revealed that the top half of patients showed robust depression improvement (69%) while the bottom half received no significant benefit (8%) by day 5. However, the bottom half showed significant improvement (36%) at two-to-four-week follow up (p = 0.001). These results suggest 5×5 accelerated rTMS treatment is as effective as once daily rTMS treatment and that the efficacy of accelerated treatment may not be accurately assessed until weeks after treatment completion.

## Introduction

Major Depressive Disorder (MDD) is the leading cause of disability and reduced productivity worldwide, which in part reflects the long and chronic course of the illness in many patients [1–4]. Antidepressant medications are highly effective for many individuals, but the initial antidepressant selected leads to remission of symptoms in less than one-third of individuals [5,6], resulting in a long process of trial-and-error with multiple medications without achieving remission.

Repetitive transcranial magnetic stimulation (rTMS) is a noninvasive neuromodulation treatment for MDD that can be highly effective even after multiple medications have failed to provide benefit [7–10]. Treatment typically involves administration of 3000 pulses of rhythmic (10 to 20 Hz) or 600 pulses of intermittent theta burst stimulation (iTBS) stimulation to the left dorsolateral prefrontal cortex (L-DLPFC; [11–13]) once daily for six weeks. However, some data indicate that up to 1800 pulses of prolonged iTBS (piTBS) is a more effective treatment than regular iTBS [14].

More recent “accelerated” approaches employ multiple treatments per day over several consecutive days to achieve response more rapidly, in as short a time as one week [15–18]. The schedule, frequency, intensity, and duration of accelerated TMS protocols vary considerably (e.g., see Holtzheimer et al., [18] and Cole et al., [16]), and the effects of frequency and duration of accelerated treatment on depression treatment outcome have not been systematically examined [19–22]. Previous studies have suggested protocols may differ regarding the degree of benefit attained and its durability [20]. Paradoxically, more intense protocols (e.g., more treatments per day, more pulses per treatment) may provide less overall clinical benefit: one study investigating the efficacy of accelerated deep TMS protocols in naturalistic clinical settings reported that 5x versus 10x daily accelerated deep TMS led to response rates of 81% versus 67.6%, respectively [23]. However, there are few studies comparing the relative efficacy, durability, and response pattern of once daily versus accelerated rTMS.

The primary goals of this study were to compare the efficacy of conventional versus an accelerated rTMS protocol in 175 patients undergoing treatment for MDD, with the accelerated protocol using five treatments per day for five days (“5×5 protocol”). We examined both immediate efficacy of both protocols as well as response patterns that emerged in the first four weeks after the completion of rTMS.

## Materials and Methods

### Patient Population

We examined data from 175 clinical patients who received rTMS for MDD at the UCLA TMS Clinical and Research Service (Los Angeles, CA, USA) from February 2023 to March 2025. This retrospective analysis was approved by the UCLA Institutional Review Board. All data were collected based on guidelines from the Declaration of Helsinki [24]. Patients underwent a structured interview to confirm the presence of a MDD diagnosis using the MINI International Diagnostic Interview [25], and all patients had at least two unsuccessful antidepressant treatment trials. Of the 175 patients in the overall sample, 135 patients received conventional rTMS consisting of at least 30 rTMS treatments (one treatment per day, five days per week, for 6 weeks; [26]). Accelerated rTMS consisted of piTBS (n = 25; [15, 27]) or individualized resonant frequency rTMS (RF, n = 15; [28]) five times per day for five consecutive days, for a total of at least 25 sessions.

### Clinical Assessments

Patients completed pre-treatment baseline depression rating scales and repeated the measures after every five rTMS sessions (i.e., weekly for conventional patients and daily for accelerated patients). The Patient Health Questionnaire-9 (PHQ-9; [29]) is a self-report depression inventory with scores ranging from 1 (minimal depression) to 27 (severe depression), and is known to be most sensitive to clinical improvement during rTMS for depression [26, 30, 31]. The Inventory of Depressive Symptomology Self-Report (IDS-SR-SR; [32, 33] measures overall depression symptoms with scores ranging from 0 (minimal depression) to 84 (severe depression). The PHQ-9 was administered at baseline and again after completion of treatment; accelerated patients completed the PHQ-9 two-to-four weeks (n = 33) after completion and conventional patients completed the PHQ-9 four weeks (n = 98) after treatment completion.

### rTMS Procedures

Treatments were applied with either the MagPro X100 (MagVenture, Inc., GA, USA), Magstim Horizon (Magstim Inc, MN, USA), or Magstim Super Rapid2 (Magstim Inc, MN, USA) TMS devices. After completing pre-treatment questionnaires, resting motor threshold (rMT) was measured by recording the minimum stimulus intensity required to elicit a motor evoked potential in the right abductor pollicis brevis muscle for at least 50% of trials of single rTMS pulses applied to the primary motor cortex.

Conventional rTMS treatment consisted of at least 30 rTMS treatment sessions with either 3000 pulses 10 Hz rTMS (*n* = 132) or 1800 pulses iTBS (*n* = 3) administered to L-DLPFC targeted with the Beam F3 method [34]. Stimulation intensity was increased to 120% rMT as tolerated over the first five treatments. Parameters were adjusted using a measurement-based care paradigm as previously described in which subjects unable to tolerate the initial stimulation or demonstrating limited improvement after 10 treatments underwent protocol adjustments to optimize response and tolerability [26, 35–41].

Patients receiving accelerated rTMS had five sessions per day (with 45 minutes between sessions) for five consecutive days (“5×5” for a total of 25 treatment sessions) utilizing either piTBS (1800 pulses) or resonant frequency (RF) rTMS. Briefly, RF-rTMS begins with a TMS-EEG “frequency mapping” procedure that involves administering trains of 40 pulses followed by an 11 second intertrain interval (ITI) at frequencies ranging between 3–20 Hz to L-DLPFC while collecting electroencephalography (EEG) data. Changes in EEG connectivity from before to after each pulse train were examined to determine each patient’s Resonant Frequency (RF), which is the stimulation frequency predicted to elicit the greatest improvement in depression scores according to a LASSO regression model [28]. Patient RFs ranged from 6–17 Hz. RF-rTMS patients received 3000 pulses per session at their RF. As above, we increased intensity to 120% rMT as tolerated.

## Data analysis

### Comparison of Baseline Demographic and Clinical Variables

All analyses were performed using R version 4.4.1 in RStudio [42]. Baseline comparisons of age, self-report biological sex, PHQ-9, and IDS-SR scores were performed between conventional and accelerated patient groups using t-tests (age, PHQ-9, and IDS-SR scores) or chi-square tests (biological sex) to ensure they were comparable prior to treatment.

#### Efficacy at End of Treatment of Conventional Versus Accelerated rTMS

Two separate linear mixed-effects models of the depression rating (one for PHQ-9 and one for IDS-SR as the outcome variable) were built to compare differences in change of depressive burden over time based on the rTMS treatment group (i.e., compare conventional and accelerated rTMS) using the lmerTest package [43] in R. Both models included timepoint (pre- vs post-rTMS) and treatment type (conventional vs accelerated) as fixed effects, a timepoint-by-treatment type interaction term, and individual participants as a random intercept. Note that “post-rTMS” was defined as PHQ-9/IDS-SR scores recorded at session 30 for the conventional patients and at session 25 for the accelerated patients, each representing completion of an initial treatment course. The Bonferroni correction (.05 / 2) was computed as a multiple comparison correction for the two models.

To assess response patterns at treatment completion, a median split was performed with the conventional and accelerated groups based on PHQ-9 median % change from baseline to the treatment session 30 for the conventional group (54.42%) and treatment session 25 for the accelerated group (39.60%), with patients greater than or equal to the median grouped as the “top half” and those with % change scores less than the median were grouped as the “bottom half.” Age and biological sex were compared between the 5×5 top and bottom halves using a t-test and chi-square test, respectively.

### Comparing piTBS and RF Accelerated Protocols

Independent-sample t-tests were conducted both for baseline PHQ-9 scores and end of treatment PHQ-9 and IDS-SR percent improvements to examine baseline equivalence and compare relative clinical efficacy between the two accelerated protocol groups, RF and piTBS.

#### Follow-Up Score Trends

Wilcoxon rank sum tests (selected here for lack of normality) were performed to compare the post-treatment follow-up PHQ-9 scores between conventional and accelerated patients. Then, comparisons were performed using the same median split approach outlined above to assess differences in the follow-up scores between the conventional and accelerated top and bottom halves. Four Wilcoxon sign-rank tests were computed to assess within-group PHQ-9 percent improvement from treatment completion to follow-up in the conventional top half, conventional bottom half, accelerated top half, and accelerated bottom half. The Bonferroni correction (.05 / 4) was performed to account for multiple comparisons among these four groups. For these analyses, four-week follow-up scores were used for conventional patients, and the last recorded score between the two- and four-week follow-up points were used for accelerated patients.

## Results

### Comparison of Baseline Demographic and Clinical Variables

There were no statistically significant differences in baseline age, sex, or depression symptom scores between the conventional and accelerated groups (Table 1).

### Efficacy at End of Treatment of Conventional Versus Accelerated rTMS

**Figure 1:**
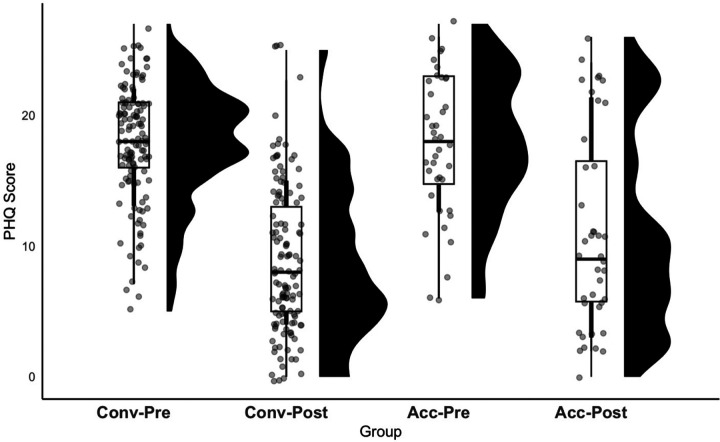
Both conventional and accelerated rTMS protocols reduced PHQ-9 scores. The two box-and-whisker plots on the left correspond to the conventional group and the two box-and-whisker plots on the right correspond to the accelerated group. The distribution of PHQ-9 scores is shown by the black “raincloud” to the right of each box-and-whsiker plot. Conv-Pre = conventional group, pre-rTMS score; Conv-Post = conventional group, post-rTMS score (S30); Acc-Pre = accelerated group, pre-rTMS score; Acc-Post = accelerated group, post-rTMS score (S25).

**Figure 2:**
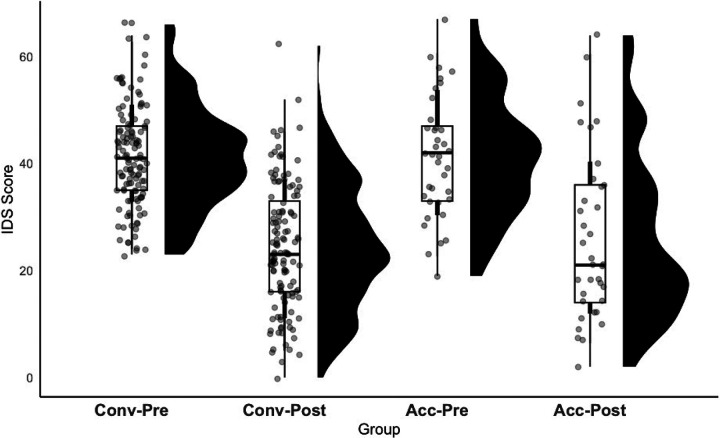
Both conventional and accelerated rTMS protocols reduced IDS-SR scores. The two box-and-whisker plots on the left correspond to the conventional group and the two box-and-whisker plots on the right correspond to the accelerated group. The distribution of PHQ-9 scores is shown by the black “raincloud” to the right of each box-and-whsiker plot. Conv-Pre = conventional group, pre-rTMS score; Conv-Post = conventional group, post-rTMS score (S30); Acc-Pre = accelerated group, pre-rTMS score; Acc-Post = accelerated group, post-rTMS score (S25).

The mixed-effects linear models indicated a significant effect of timepoint (pre versus post TMS) on the PHQ-9 and IDS-SR, indicating both the conventional and accelerated protocols reduced depression scores (Figs. 1 and 2). The main effect of protocol was not significant for either of the models. There was a statistically significant interaction term for the PHQ-9 model (*p* = 0.038), indicating that the reduction in scores from pre- to post-rTMS for the conventional group (pre-rTMS PHQ-9 mean = 17.83 ± 4.44; post-rTMS PHQ-9 mean = 8.97 ± 5.79) differed from the accelerated group (pre-rTMS PHQ-9 mean = 17.68 ± 5.47; post-rTMS PHQ-9 mean = 10.98 ± 7.68), but this effect did not survive multiple comparison correction via the Bonferroni correction (.05 / 2), yielding a significance threshold of .025 or a corrected PHQ-9 model interaction term *p*-value of 0.07. The interaction term for the IDS-SR model was not statistically significant (*p* = 0.492; Table 2).

The median split at treatment completion revealed the top half of subjects in both the conventional and accelerated groups showed substantial and comparable degrees of mean improvement (74.7% and 68.7%, respectively). Subjects in the bottom half of the conventional group were partial responders (27.6%), while those in the bottom half of the 5×5 group showed negligible improvement (7.93%). There were no statistically significant differences in age [t(37.85) = 0.76472, *p* = 0.4492] or biological sex [χ^2^(1) = 0.101, *p* = 0.751] between the 5×5 top and bottom halves.

### Comparing piTBS and RF Accelerated Protocols

RF accelerated patients had average higher pre-treatment baseline PHQ-9 scores (*mean* = 20.27) compared to the piTBS patients (*mean* = 16.12), *t*(34.18) = 2.591, *p* = 0.01397. There were no statistically significant differences in the percent improvement in PHQ-9 or IDS-SR between RF and piTBS accelerated patients (*p* > .05; Table 3).

### Efficacy at Follow-Up of Conventional Versus Accelerated rTMS

**Figure 3:**
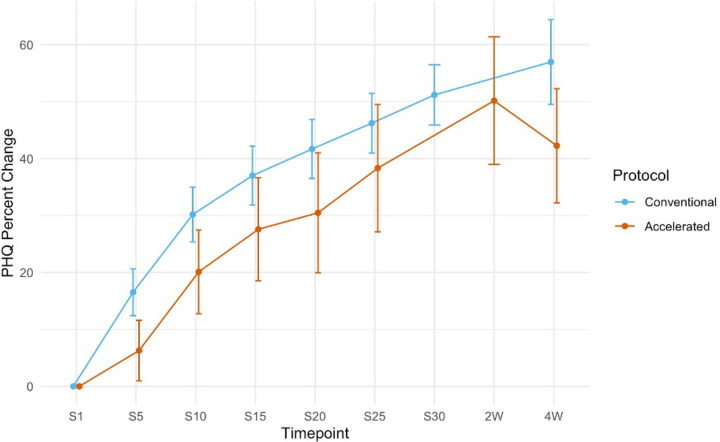
PHQ-9 scores from session 1 to treatment completion (session 25 for accelerated patients and session 30 for conventional patients. Follow up PHQ-9 scores between the conventional and accelerated patients at 2 (accelerated) and 4 (conventional and accelerated) weeks after the completion of rTMS can be observed. Standard error bars are shown for each data point.

The PHQ-9 scores of conventional and accelerated patients out to the four-week follow-up point displayed similar PHQ-9% improvement (Fig. 3). The conventional group had a greater four-week follow-up PHQ-9% improvement relative to baseline (56.95%) compared to the two-to-four-week follow-up measures for the accelerated group (45.75%), W = 1933.5, *p* = 0.01468.

The follow-up score median split analyses were performed with the Bonferroni correction to account for multiple comparisons between these four groups, yielding an adjusted significance threshold of 0.0125. Among the four outcome groups, only the accelerated bottom-half group showed a statistically significant increase in PHQ-9% improvement from treatment completion to follow-up (V = 0, *p* = 0.001). The other three groups did not exhibit significant changes with the adjusted significance threshold: accelerated top half (V = 81, *p* = 0.014), conventional top half (V = 227, *p* = 0.231), and conventional bottom half (V = 241, *p* = 0.0382).

## Discussion

These results indicate that both conventional and 5×5 rTMS protocols provide significant clinical benefit as indicated by overall degree of symptom improvement, with the 5×5 protocols providing relief much more rapidly than conventional once-daily treatment. There were no significant differences in MDD symptom improvement between conventional and 5×5 rTMS protocols following multiple comparison correction. The degree of benefit for the 5×5 subjects in this study, measured by average response rates ranging from 38–50% between treatment completion and the four-week follow-up point, is comparable to what has been reported overall for previous studies of accelerated treatment (52.2%; [20]), despite the fact that subjects in this study received fewer sessions (25) than in many other protocols. This is the first report on use of RF accelerated treatment for MDD, and there was no significant difference in degree of benefit from piTBS, even though the RF subjects had more severe average baseline depression scores.

The results of the median split analysis highlight the importance of extending symptom assessment when investigating the efficacy of accelerated rTMS protocols. The top half subjects in this study showed rapid and robust benefit while the bottom half had negligible benefit (69% vs. 8% improvement by day 5, respectively). This pattern is similar to what has been reported with conventional treatment [44], and at the end of acute treatment one could have concluded that the bottom half of patients were non-responders or had simply not had enough sessions. At two-to-four-week follow-up, however, the bottom half of 5×5 subjects showed a statistically significant mean depression improvement of 36%, with many bottom-half accelerated patients being comparable to the top-half accelerated and conventional patients. No other subgroup showed such substantial improvement up to one month of follow up.

These findings indicate that assessing efficacy of accelerated treatment is complex and that accelerated rTMS treatment efficacy should be assessed both at the end of acute treatment and at follow-up timepoints. At the end of acute treatment (five days), only half our sample derived significant benefit. The bottom half of the 5×5 patients in this study failed to benefit from treatment at day 5, but were comparable to the top half of 5×5 patients 14–28 days later. These results indicate that there is a delayed response in accelerated rTMS treatment and that simple pre-post depression measures at single timepoints may fail to capture such delayed responses in some patients [45, 46].

Additionally, although the accelerated protocol cleared by the FDA utilized 50 sessions over five days [16], our results suggest fewer sessions may be sufficient to elicit a response. This is an important consideration because delivering 10 sessions per day of rTMS may not be practical in clinical settings. This is in line with previous work indicating that clinical benefit can be attained with as few as 15 treatments over two days [18], suggesting that even the 25 sessions utilized here may be longer than necessary for some patients. Another report examining accelerated TMS delivered using a H coil (deep TMS) reported that a greater number of sessions over more days yielded lesser degrees of benefit [23], so it is not clear that more treatments delivered acutely would have yielded better outcomes.

Importantly, these results highlight the need to assess how conventional and accelerated rTMS protocols differ in delivering enduring benefit. Conventional rTMS treatment is known to deliver durable benefit, with 50% of patients maintaining their improvement over one year (although some retreatment is necessary for some patients; [47]). Past accelerated rTMS research has not always included follow-up measures after the treatment conclusion [48]. There was a statistically significant difference in follow-up scores between the accelerated and conventional groups suggesting that the conventional group had a greater PHQ-9% improvement score (57%) compared to the accelerated group (46%) at the four versus two-to-four week follow-up points, respectively. In our accelerated sample, those with minimal benefit showed substantially greater benefit at follow up, while those with the greatest acute benefit showed a small numeric but not statistically significant loss of benefit following multiple comparison correction. This worsening is in line with previous studies reporting that although accelerated TMS can lead to rapid improvement, benefits can be short-lived for many patients [16, 20]. One recent study showed that more than half of patients who entered remission acutely with an intense treatment schedule (50 sessions over five days) relapsed within 12 weeks [49]. Rather than providing lengthy, intensive treatment for all patients initially (i.e., 50 sessions), it is possible that a more effective approach to extend treatment benefit would be to administer an additional day of treatment at the two-to-four week follow up point. Such additional sessions might be useful both for gaining greater benefit or extending treatment benefits for a longer period, and this possibility should be further explored in additional investigations.

### Limitations and Future Directions

These results must be interpreted in the context of several limitations. Data were collected from patients in a naturalistic clinical setting [26, 27, 35] in which subjects were not randomly and prospectively assigned to conventional or accelerated 5×5 groups treatment, and there was no sham TMS condition. There was no experimental control for psychiatric/medical comorbidities, socioeconomic status, or demographic factors, although outcome appeared to be unrelated to age, gender, or depression severity. 5×5 subjects in this study were treated with either piTBS or rTMS at their individual RFs (which ranged from 6–17 Hz); while these two approaches yielded comparable results, treatment assignment to these groups also was not random, but instead was determined by which subjects consented to inclusion in a study of the efficacy of RF treatment. Attrition occurred in the two-to-four-week PHQ-9 follow-up measures, so that comparisons beyond the treatment end points (after treatment 30 for the conventional group and after treatment 25 for the accelerated group) should be interpreted conservatively. Additionally, conventional patients received five more rTMS sessions than the accelerated patients, and the follow-up analyses were performed with non-parametric statistics, which should increase caution when interpreting the results [50].

Future investigations should explore the differential efficacy of conventional and accelerated rTMS protocols in improving other depression symptoms (e.g., anhedonia or sleep disturbances) while engaging in random sampling procedures and controlling for additional individual factors. Future work should continue to explore the degree to which treatment effects persist beyond the termination of the treatment protocol between conventional and accelerated approaches [48]. The naturalistic clinical setting and the measurement-based care paradigm generated variability in terms of the stimulator models, initial stimulation parameters, parameter adjustments throughout treatment, supplemental or continued outpatient psychiatric care received during rTMS. It is not known how these factors impacted the results, and the influence of these parameters on treatment outcomes should be explored in future studies. Related, future investigations should explore how different targeting methods (e.g., heuristic versus structural MRI versus functional MRI) impact treatment outcomes. Finally, the identification of biomarkers (e.g., functional neuroimaging resting-state networks) that predict response to conventional versus accelerated rTMS protocols will be an important step in future work.

## Conclusions

These findings suggest that conventional once-daily and 5×5 accelerated rTMS have similar efficacy in improving depression scores. Furthermore, we reported that clinical improvement can continue to be observed two and four weeks after the conclusion of treatment, notably in patients who did not initially respond to 5×5 accelerated treatment, highlighting the importance of assessing treatment efficacy beyond the completion of the treatment protocol. Although future studies with additional controls are needed, these results are a step towards increased personalization in selecting rTMS protocols that are likely to work best for each individual patient in a clinical setting.

## Figures and Tables

**Figure 4:**
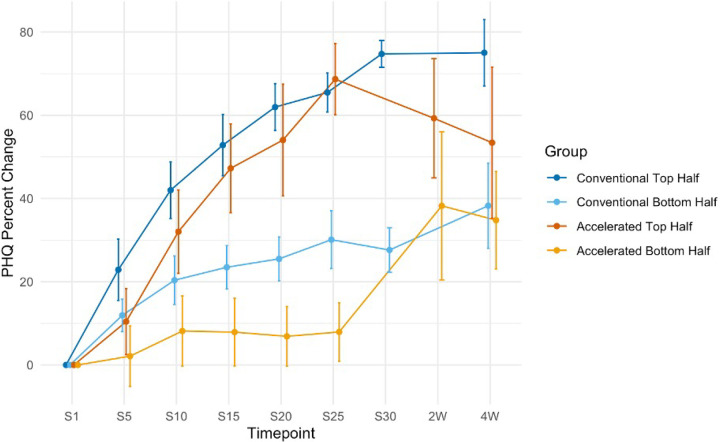
Median split trajectories for the conventional and accelerated top and bottom half patients. The accelerated bottom half patients display significant improvement following treatment completion. Standard error bars are shown for each data point.

**Table 1:** Baseline demographics and clinical variables. There were no statistically significant differences in baseline age, biological sex, PHQ-9, or IDS-SR.

	Conventional	Accelerated	Statistical Test
Age (years)	46.69	49.90	*t*(58.157) = −1.0116, *p* = 0.3159;
Biological Sex	female: 71, male: 62, non-binary: 2	female: 22, male: 18, non-binary: 0	χ^2^(2) = 0.632, *p* = 0.729;
PHQ-9	17.83	17.68	*t*(56.398) = 0.16483, *p* = 0.8697;
IDS-SR	41.35	41.30	*t*(52.359) = 0.024329, *p* = 0.9807

**Table 2:** Mixed-effects linear models predicting the outcome variables (Model column) with Treatment (conventional and accelerated), Pre/Post (Pre = S1 and Post = S25 for accelerated patients and S30 for conventional patients), and Treatment × Pre/Post (interaction term) as fixed effects, and participants as random effects.

Model	Fixed Effect	Estimate +/− Standard Error	*t*-value	*p*-value
PHQ-9	Treatment	−0.1985 +/− 1.0081	−0.197	0.8441
	Pre/Post	−8.8947 +/− 0.5211	−17.071	< 2e-16
	Treatment × Pre/Post	2.1947 +/− 1.0478	2.095	0.0378
IDS-SR	Treatment	−0.1636 +/− 2.1383	−0.077	0.939
	Pre/Post	−17.0598 +/− 1.0652	−16.015	< 2e-16
	Treatment × Pre/Post	1.5273 +/− 2.218	0.689	0.492

**Table 3:** Independent-samples t-tests were computed to compare percent change in each of the outcome variables between the RF and piTBS accelerated patients.

Variable	RF Average % Change	piTBS Average % Change	*t*-value	*p*-value
PHQ-9	39.19397	37.77843	0.12066	0.9048
IDS-SR	34.47394	42.24611	−0.80382	0.4285
